# Supplementary material for: Post-exertion oxygen saturation as a prognostic factor for adverse outcome in patients attending the emergency department with suspected COVID-19: a substudy of the PRIEST observational cohort study
Source: Emerg Med J. 2020 Dec 3;38(2):88–93. doi: 10.1136/emermed-2020-210528 (PMC7716294; doi:10.1136/emermed-2020-210528)

Supplemental Table S1: Characteristics of the PRIEST cohort and the cohort included in this analysis

Characteristic		All (n=22445)	With post-exertion oxygen saturation (n=817)
Sex			
	Male	11034 (49.2%)	369 (45.2%)
	Female	11200 (49.9%)	442 (54.1%)
	Missing	211 (0.9%)	6 (0.7%)
Ethnicity			
	Uk\Irish\other white	15198 (67.7%)	475 (58.1%)
	Asian	1150 (5.1%)	67 (8.2%)
	Black/African/Caribbean	692 (3.1%)	47 (5.8%)
	Mixed/multiple ethnic groups	328 (1.5%)	18 (2.2%)
	Other	570 (2.5%)	47 (5.8%)
	Missing	4507 (20.1%)	163 (20.0%)
Performance status			
	Unrestricted normal activity	11917 (53.1%)	643 (78.7%)
	Limited strenuous activity, can do light	2393 (10.7%)	83 (10.2%)
	Limited activity, can self care	2790 (12.4%)	36 (4.4%)
	Limited self care	2662 (11.9%)	11 (1.3%)
	Bed/chair bound, no self care	1510 (6.7%)	0 (0.0%)
	Missing	1173 (5.2%)	44 (5.4%)
Age			
	N (%)	22438 (100.0%)	817 (100.0%)
	Mean (SD)	58.4 (24.2)	48.4 (16.1)
	Median (IQR)	62.0 (43.0, 78.0)	47.0 (36.0, 59.0)
Respiratory rate			
	N (%)	21843 (97.3%)	796 (97.4%)
	Mean (SD)	23.9 (7.7)	20.8 (5.3)
	Median (IQR)	22.0 (18.0, 28.0)	20.0 (18.0, 23.0)
Pulse Rate			
	N (%)	21966 (97.9%)	809 (99.0%)
	Mean (SD)	97.8 (24.5)	90.6 (18.1)
	Median (IQR)	95.0 (81.0, 111.0)	90.0 (78.0, 102.0)
Temperature			
	N (%)	21740 (96.9%)	799 (97.8%)
	Mean (SD)	37.2 (1.1)	37.0 (0.8)
	Median (IQR)	37.0 (36.5, 37.9)	36.8 (36.5, 37.3)
Systolic BP			
	N (%)	20697 (92.2%)	793 (97.1%)

Mean (SD)	134.2 (25.0)	136.5 (21.3)
Median (IQR)	132.0 (117.0, 149.0)	134.0 (122.0, 148.0)
Diastolic BP		
N (%)	20600 (91.8%)	788 (96.5%)
Mean (SD)	78.0 (16.2)	82.6 (12.9)
Median (IQR)	78.0 (68.0, 88.0)	82.0 (74.5, 90.0)
Oxygen saturation		
N (%)	22154 (98.7%)	813 (99.5%)
Mean (SD)	94.9 (6.6)	97.0 (2.4)
Median (IQR)	96.0 (94.0, 98.0)	97.0 (96.0, 99.0)
Medical History		
No Chronic disease	7077 (31.5%)	406 (49.7%)
Heart Disease	4723 (21.0%)	66 (8.1%)
Renal impairment	1944 (8.7%)	27 (3.3%)
Steroid therapy	564 (2.5%)	13 (1.6%)
Asthma	3492 (15.6%)	135 (16.5%)
Diabetes	4132 (18.4%)	73 (8.9%)
Active malignancy	1124 (5.0%)	14 (1.7%)
Immunosuppression	646 (2.9%)	30 (3.7%)
Other chronic lung disease	3795 (16.9%)	81 (9.9%)
Hypertension	6439 (28.7%)	155 (19.0%)
Medical history missing	1104 (4.9%)	50 (6.1%)
Adverse outcome		
Any	4638 (20.7%)	30 (3.7%)
Death	3251 (14.5%)	8 (1.0%)
Respiratory support	1962 (8.7%)	22 (2.7%)
Cardiovascular support	525 (2.3%)	5 (0.6%)
Renal support	220 (1.0%)	4 (0.5%)

Supplemental Table S2: Accuracy of baseline oxygen saturation at a range of thresholds for positivity, primary analysis (N=813)

Threshold	Sensitivity (95% CI)	Specificity (95% CI)	Positive likelihood ratio (95% CI)	Negative likelihood ratio (95% CI)
<=99	96.7 (82.8 to 99.9)	14.9 (12.5 to 17.6)	1.14 (1.06 to 1.23)	0.22 (0.03 to 1.52)
<=98	90.0 (73.5 to 97.9)	28.6 (25.5 to 31.9)	1.26 (1.11 to 1.43)	0.35 (0.12 to 1.03)
<=97	76.7 (57.7 to 90.1)	49.3 (45.7 to 52.9)	1.51 (1.23 to 1.86)	0.47 (0.24 to 0.90)
<=96	70.0 (50.6 to 85.3)	66.2 (62.7 to 69.5)	2.07 (1.61 to 2.67)	0.45 (0.26 to 0.78)
<=95	53.3 (34.3 to 71.7)	78.3 (75.2 to 81.1)	2.46 (1.72 to 3.53)	0.60 (0.41 to 0.88)
<=94	43.3 (25.5 to 62.6)	88.5 (86.1 to 90.7)	3.77 (2.40 to 5.93)	0.64 (0.47 to 0.88)
<=93	33.3 (17.3 to 52.8)	93.9 (92.0 to 95.4)	5.44 (3.06 to 9.67)	0.71 (0.55 to 0.92)
<=92	30.0 (14.7 to 49.4)	95.5 (93.8 to 96.9)	6.71 (3.55 to 12.67)	0.73 (0.58 to 0.92)
<=91	20.0 (7.7 to 38.6)	97.7 (96.4 to 98.6)	8.70 (3.72 to 20.33)	0.82 (0.69 to 0.98)
<=90	16.7 (5.6 to 34.7)	98.5 (97.3 to 99.2)	10.87 (4.09 to 28.89	0.85 (0.72 to 1.00)

Supplemental Table S3: Accuracy of post-exertion oxygen saturation at a range of thresholds for positivity, primary analysis (N=817)

Threshold	Sensitivity (95% CI)	Specificity (95% CI)	Positive likelihood ratio (95% CI)	Negative likelihood ratio (95% CI)
<=99	100 (88.4 to 100)	9.8 (7.8 to 12.1)	1.11*	0.00*
<=98	96.7 (82.8 to 99.9)	20.5 (17.7 to 23.4)	1.22 (1.13 to 1.32)	0.16 (0.02 to 1.10)
<=97	93.3 (77.9 to 99.2)	36.7 (33.3 to 40.2)	1.47 (1.32 to 1.64)	0.18 (0.05 to 0.69)
<=96	86.7 (69.3 to 96.2)	48.4 (44.9 to 52.0)	1.68 (1.44 to 1.96)	0.28 (0.11 to 0.70)
<=95	66.7 (47.2 to 82.7)	58.2 (54.7 to 61.7)	1.59 (1.22 to 2.07)	0.57 (0.34 to 0.95)
<=94	56.7 (37.4 to 74.5)	66.8 (63.4 to 70.1)	1.71 (1.23 to 2.37)	0.65 (0.43 to 0.98)
<=93	56.7 (37.4 to 74.5)	73.4 (70.2 to 76.5)	2.13 (1.53 to 2.97)	0.59 (0.39 to 0.89)
<=92	53.3 (34.3 to 71.7)	76.2 (73.1 to 79.2)	2.24 (1.57 to 3.20)	0.61 (0.42 to 0.90)
<=91	43.3 (25.5 to 62.6)	81.4 (78.6 to 84.1)	2.34 (1.52 to 3.61)	0.70 (0.51 to 0.96)
<=90	36.7 (19.9 to 56.1)	86.0 (83.4 to 88.4)	2.62 (1.59 to 4.32)	0.74 (0.56 to 0.97)

*Unable to calculate confidence interval

Table S4: Accuracy of baseline oxygen saturation at a range of thresholds for positivity, secondary analysis (N=652)

Threshold	Sensitivity (95% CI)	Specificity (95% CI)	Positive likelihood ratio (95% CI)	Negative likelihood ratio (95% CI)
<=99	93.8 (69.8 to 99.8)	15.1 (12.4 to 18.1)	1.10 (0.97 to 1.25)	0.41 (0.06 to 2.76)
<=98	81.3 (54.4 to 96.0)	30.0 (26.5 to 33.8)	1.16 (0.91 to 1.48)	0.62 (0.22 to 1.73)
<=97	68.8 (41.3 to 89.0)	52.8 (48.9 to 56.8)	1.46 (1.04 to 2.05)	0.59 (0.28 to 1.22)
<=96	56.3 (29.9 to 80.2)	70.9 (67.2 to 74.4)	1.93 (1.23 to 3.02)	0.62 (0.35 to 1.08)
<=95	25.0 (7.3 to 52.4)	84.3 (81.2 to 87.0)	1.59 (0.67 to 3.79)	0.89 (0.67 to 1.18)
<=94	6.3 (0.2 to 30.2)	95.1 (93.2 to 96.7)	1.28 (0.19 to 8.81)	0.99 (0.87 to 1.12)

Table S5: Accuracy of post-exertion oxygen saturation at a range of thresholds for positivity, secondary analysis

Threshold	Sensitivity (95% CI)	Specificity (95% CI)	Positive likelihood ratio (95% CI)	Negative likelihood ratio (95% CI)
<=99	100.0 (79.4 to 100.0)	10.3 (8.1 to 13.0)	1.12*	0.00*
<=98	93.8 (69.8 to 99.8)	21.8 (18.6 to 25.2)	1.20 (1.05 to 1.37)	0.29 (0.04 to 1.95)
<=97	93.8 (69.8 to 99.8)	39.1 (35.3 to 43.0)	1.54 (1.34 to 1.77)	0.16 (0.02 to 1.07)
<=96	93.8 (69.8 to 99.8)	52.1 (48.2 to 56.0)	1.96 (1.69 to 2.28)	0.12 (0.02 to 0.80)
<=95	62.5 (35.4 to 84.8)	62.1 (58.2 to 65.9)	1.65 (1.11 to 2.44)	0.60 (0.32 to 1.13)
<=94	50.0 (24.7 to 75.3)	71.2 (67.5 to 74.7)	1.74 (1.05 to 2.88)	0.70 (0.43 to 1.15)
<=93	50.0 (24.7 to 75.3)	77.9 (74.5 to 81.1)	2.27 (1.36 to 3.78)	0.64 (0.39 to 1.05)
<=92	50.0 (24.7 to 75.3)	80.9 (77.6 to 83.9)	2.62 (1.56 to 4.39)	0.62 (0.38 to 1.01)
<=91	37.5 (15.2 to 64.6)	85.0 (82.0 to 87.7)	2.50 (1.29 to 4.83)	0.74 (0.51 to 1.08)
<=90	37.5 (15.2 to 64.6)	89.5 (86.9 to 91.8)	3.58 (1.83 to 7.01)	0.70 (0.48 to 1.02)

*Unable to calculate confidence interval

Figure S1: Flow of patients through the study

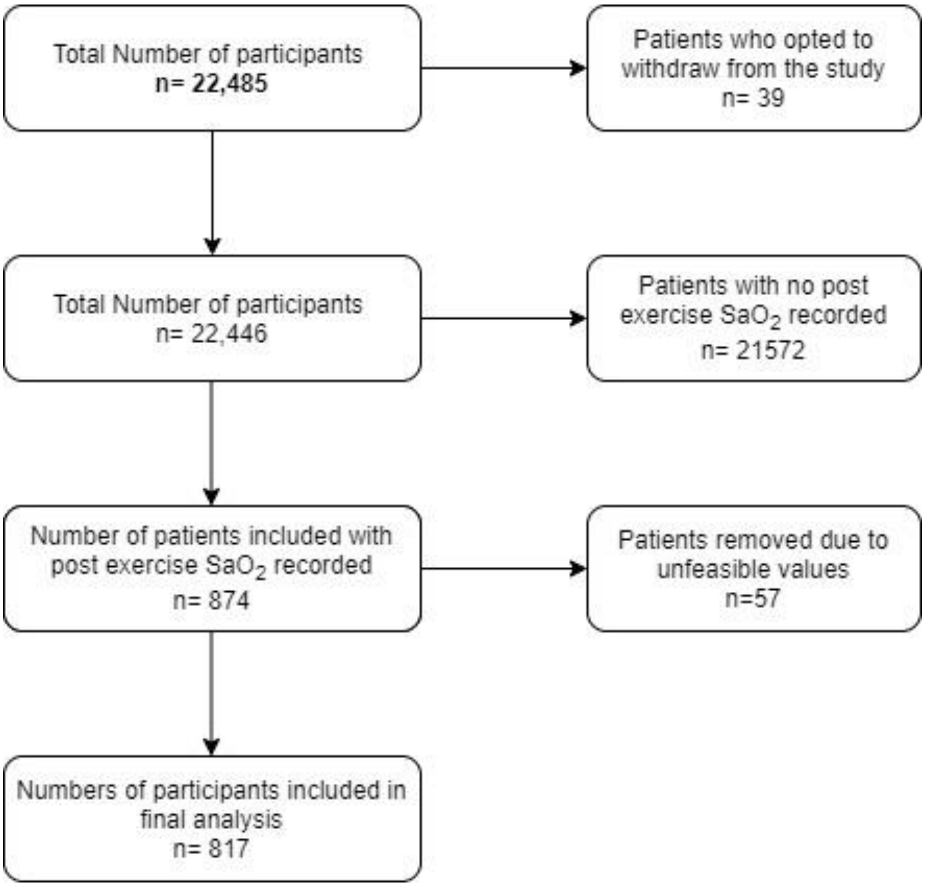

Figure S2: Histograms comparing baseline oxygen saturation between patients with and without adverse outcome (N=813)

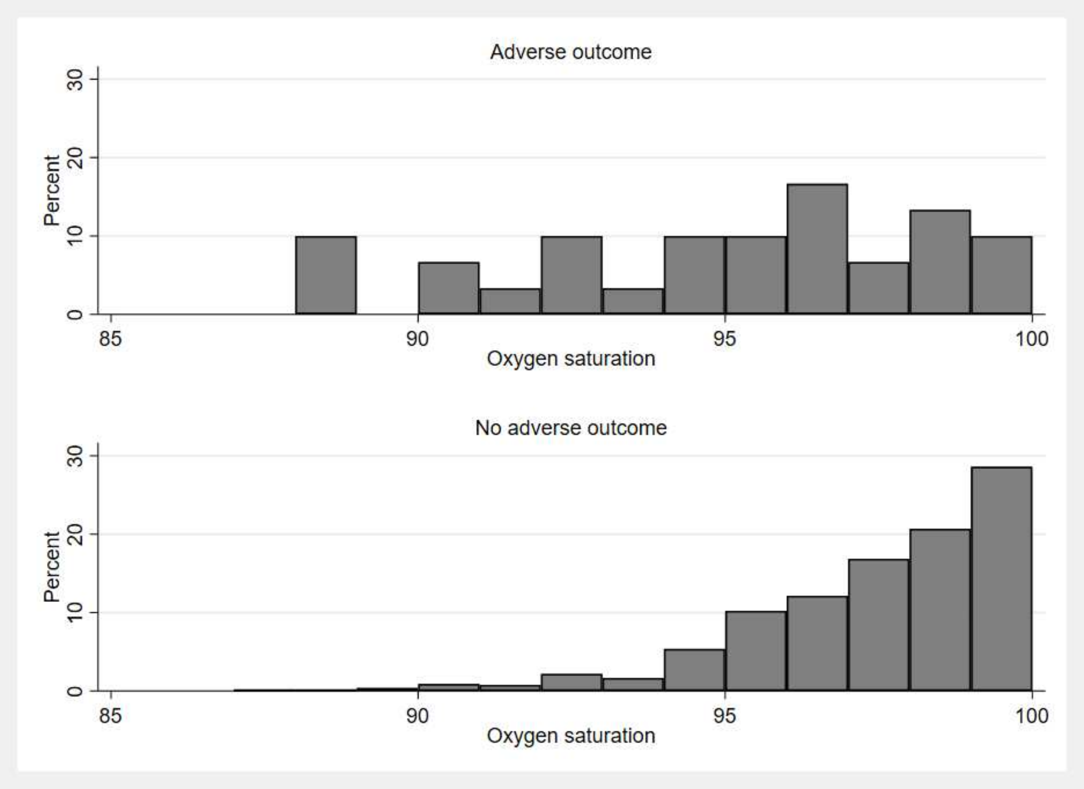

Figure S3: Histograms comparing post-exertion oxygen saturation between patients with and without adverse outcome (N=817)

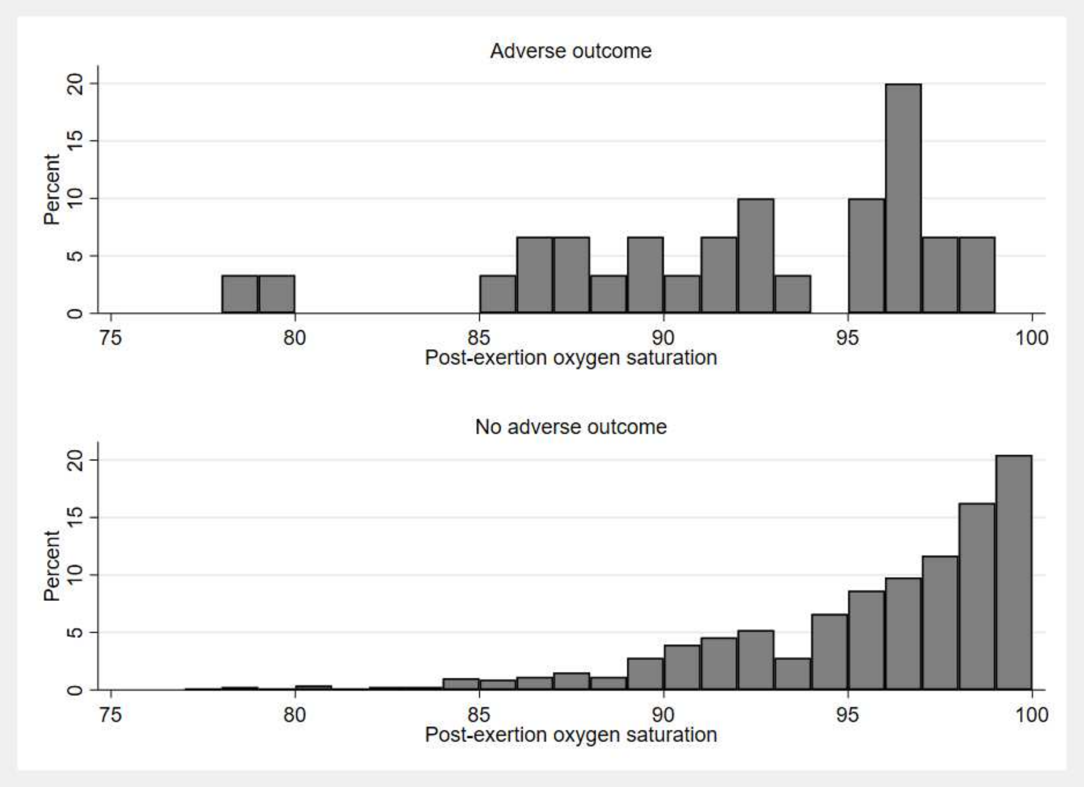

Supplement: Supplementary data [file emermed-2020-210528supp001.pdf]
