# Supplementary material for: Post-exertion oxygen saturation as a prognostic factor for adverse outcome in patients attending the emergency department with suspected COVID-19: a substudy of the PRIEST observational cohort study
Source: Emerg Med J. 2020 Dec 3;38(2):88–93. doi: 10.1136/emermed-2020-210528 (PMC7716294; doi:10.1136/emermed-2020-210528)
Supplement: Supplementary data [file emermed-2020-210528supp002.pdf]

Appendix 1: The PRIEST study research team

**Name**	**Study Role**	**Role**	**Affiliation**
Ben Thomas	Study manager (lead)	Study Manager	University of Sheffield
Katie Biggs	CTRU over sight	Assistant Director	University of Sheffield
Steve Goodacre	Chief Investigator	Professor of Emergency Medicine	University of Sheffield
Carl Marincowitz	Clinical co-investigator	Clinical Lecturer in Emergency Medicine	University of Sheffield
Ellen Lee	Senior statistician	Statistician	University of Sheffield
Laura Sutton	Statistician	Statistician/Research Associate	University of Sheffield
Matthew Burnsall	Statistician	Statistician	University of Sheffield
Mike Bradburn	Senior Statistician	Senior Medical Statistician	University of Sheffield
Simon Waterhouse	Data Management	Lead Data Specialist	University of Sheffield
Richard Simmonds	Data Management	Data Management/Information Systems Officer	University of Sheffield
Jose Schutter	Research Assistant	Research Assistant	University of Sheffield
Sarah Connelly	Research Assistant	Research Assistant	University of Sheffield
Elena Sheldon	Research Assistant	Research Assistant	University of Sheffield
Jamie Hall	Research Assistant	Research Assistant	University of Sheffield
Emma Young	Research Assistant	Research Assistant	University of Sheffield
Ian Maconochie	Project Management Group, paediatric emergency medicine	Consultant in Paediatric Emergency Medicine / Associate Medical Director	Imperial College Healthcare NHS Trust

Andrew Lee	Project Management Group, public health	Reader of Global Public Health	University of Sheffield
Darren Walter	Project Management Group, emergency medicine	Clinical Senior Lecturer / Consultant in Emergency Medicine	Manchester University NHS Foundation Trust
Andrew Bentley	Project Management Group, critical care and respiratory medicine	Consultant in ICM & Respiratory Medicine	Manchester University NHS Foundation Trust, Wythenshawe Hospital
Chris Fitzimmons	Project Management Group, paediatric emergency medicine	Consultant in Paediatric Emergency Medicine	Sheffield Children's NHS Foundation Trust
Fiona Lecky	Project Management Group, emergency medicine	Clinical Professor in Emergency Medicine	University of Sheffield
Tim Harris	Project Management Group, emergency medicine	Professor of Emergency Medicine	Barts Health NHS Trust
Kirsty Challen	Project Management Group, emergency medicine	Consultant in Emergency Medicine	Lancashire Teaching Hospitals NHS Foundation Trust
