# Supplementary material for: Post-exertion oxygen saturation as a prognostic factor for adverse outcome in patients attending the emergency department with suspected COVID-19: a substudy of the PRIEST observational cohort study
Source: Emerg Med J. 2020 Dec 3;38(2):88–93. doi: 10.1136/emermed-2020-210528 (PMC7716294; doi:10.1136/emermed-2020-210528)
Supplement: Supplementary data [file emermed-2020-210528supp003.pdf]

Appendix 2: PRIEST study steering committee

Title	First Name	Last Name	Job Title	Name of employing institution, and any institutions where this nominee holds an Honorary Contract	Membership Type:	Independent	Expertise
Mrs	Shan	Bennett	PPI		PPI Member	Yes	PPI, Sheffield Emergency Care Forum
Prof (Associate Professor)	Paul	Baxter	Senior Lecturer in Biostatistics	University of Leeds. Honorary contract with Leeds Teaching Hospitals NHS Trust.	Member	Yes	Biostatistics
Prof	Tim	Coats	Professor of Emergency Medicine	University of Leicester	Chair	Yes	Clinician Emergency Medicine Research
Mrs	Enid	Hirst	Co-ordinator of Sheffield Emergency Care Forum (PPI)		PPI Member	Yes	PPI, Sheffield Emergency Care Forum
Mrs	Beryl	Darlison	PPI		PPI Member	Yes	PPI, Sheffield Emergency Care Forum
Dr	Kavin	Smith	Deputy Director Healthcare Public Health England, Yorkshire and the Humber (Replaced Will Morton as the PHE representative)	Public Health England	Member	Yes	Public health
Dr	Will	Morton	Consultant in Health Protection at Public Health England	Public Health England. Honorary contract with the University of Manchester.	Member	Yes	Health protection specialist
Dr	Nazir	Lone	Senior Clinical Lecturer in Critical Care	The University of Edinburgh, Honorary	Member	No	Cliniciain Critical Care, Critical Care

				Consultant in Critical Care at the Royal Infirmary of Edinburgh.			Epidemiology
Dr	Graham	McClelland	Research paramedic	North East Ambulance Service NHS Trust	Member	Yes	Clinician Paramedic
Prof	Steve	Goodacre	PRIEST CI	The University of Sheffield	Member	No	Clinician Emergency Medicine Research
Mrs	Rachel	Robinson	Chief Nurse	Integrated Care 24 Ltd	Member	Yes	Clinician, 111 Knowledge
Dr	Mathew	Beattie	Medical Director North East Ambulance Service Foundation Trust	North East Ambulance Service Foundation Trust	Member	Yes	Clinician, North East Ambulance Service
