# Supplementary material for: Post-exertion oxygen saturation as a prognostic factor for adverse outcome in patients attending the emergency department with suspected COVID-19: a substudy of the PRIEST observational cohort study
Source: Emerg Med J. 2020 Dec 3;38(2):88–93. doi: 10.1136/emermed-2020-210528 (PMC7716294; doi:10.1136/emermed-2020-210528)
Supplement: Supplementary data [file emermed-2020-210528supp004.pdf]

Site team acknowledgements

Anna Wilson
Bethan Holroyd-Hind
Chris Fitzsimmons
Julie Morcombe
Mrs Claire Brookes
Mrs Heidi Redfearn
Dr Ben Bloom
Imogen Skene
Raine Astin-Chamberlain
Laura Barman
Janine Mallison
Lisa Baldwin
Anneli James
Lucy Newton
Mark D Lyttle
Rebecca Hoskins
Sally Melson
Bristol Clinical Trials Team
Mrs Emma Storr
Mr Martin Walton
Dr Felix Wood
Annie Rose RGN
Francesca Wright RGN
Aarzoo Khan
Liza Keating
Emma Craig
Elizabeth Taylor
Andrew Rees MBA RICR
Simon Sharpe
Heather Sellers
Dr Tanya de Weymarn
Gloucestershire Cancer Clinical Trials Team
Jagtar Pooni
Sara Simmons
Dani Steward
Dr Adrian Marsh
Sr Mandy Carnahan
Sr Lucy Price
Dr Mark Harrison
Rebecca Emmonds
Jane Luke
Northumbria Clinical Trials Team
Mrs Katrina Parkinson
Miss Georgia Thomasson
Mrs Alda Remegoso
Bernard Hadebe
Arianna Bellini
Chloe Lyons
Tracy Marsden
Mr Paul Brittain
Joanne Galliford
Prisca Gondo
Paula Harman
Melanie Darwent
Ross Downes
Sally Beer
Dr Jennifer Lockwood
Harrogate Research team
Alasdair Gray
Polly Black
Amanda Lyle
Yvonne Lester
Dane Goodere-Bennett
Dr Huw Steven Jenkins
Dr John Wright
Kimberley Webster
Professor Richard Body
Dr Eloise Cook
Non-clinical COVID-19 Research Delivery Team at Manchester
Abdo Sattout
Melanie Harrison
Sarah Stevenson
Dr Adrian Boyle
Susie Hardwick
Debbie Read
Frank Coffey
Megan Meredith
Helen Navarra
Mrs Judith Ratcliff
Fiona Thompson
Amanda Adamson
Dr Gareth Hampton
Dr Sarah Wilson
Mrs Joana Da Rocha
Dr Charlotte Griffiths
Dr Nam Tong
Mrs Tracy Fuller
Mrs Hannah Bloxham
Alastair Richards
Debra Barnett
Lindianne Aitken

Suzannah Pegler
Maggie Walton
Tim Slade
Fleur Cantle
Hannah Cotton
Maeve Cockrell
Jessica Law
Ava Williams
Janet Mills
Janice Birt
Cassandra Gleeson
Dr Recebba Macfarlane
Mrs Lisa Evans
Ms Eloise Van Vuren
Dr Amelia Gruber
Dr Ignacio Cardona
Laura O'Rourke
Julie Quigley
Mohammad Zubair Ahmad
Daniella Hydes
Suzanne Mason
Mishel Cunningham
Nicola Lancaster
Amanda Cowton
Sarah Clark
Jane Varin
Karl Ward
Ella Sykes
Heather Jarman
Desislava Baramova
Marta Pizzorusso
Dr Sarah Essex
Mrs Andrea Watson
Mr Craig Mower
Sara Bennett
Judith Bell
Abigail Pemberton
Dr Jill Woodhead
Sherwood Forest Clinical Trials Team
Dr Amber Nocher
Dr Henrietta Morton King
Mrs Jo-Ann Taylor
Dr Shayma Habeeb
Wojciech Sawicki
Kate Martin
Nicola Charnley
Mr Matthew Edward Ryan
Dr Shrouk Messahel
Dr Daniel B Hawcutt
Miss Laura Purandare
Mr Daniel Griffiths
Miss Rebecca Miln
Robert Hull
Laura Robertson
Michaela Sutherland
Bolton Clinical Trials Team
Christine Dixon
Ellen Jessup-Dunton
Reina Layug
Dr. Rajendar Garlapati
Farzana Masters
Yvonne Grimes
Joseph Dykes
Katharine Gantert
Favour Chukwunonyerem
Dilara Arslan
