# Supplementary material for: Post-exertion oxygen saturation as a prognostic factor for adverse outcome in patients attending the emergency department with suspected COVID-19: a substudy of the PRIEST observational cohort study
Source: Emerg Med J. 2020 Dec 3;38(2):88–93. doi: 10.1136/emermed-2020-210528 (PMC7716294; doi:10.1136/emermed-2020-210528)
Supplement: Supplementary data [file emermed-2020-210528supp005.pdf]

Additional CTRU Staff Acknowledgements

Marie Hyslop

Dan Beever

Samuel Keating

Kerry Wilson

Heather Dakin

Edwin Burkinshaw

Kirsty Pemberton

Tim Chater

Chris Turtle

Emily Turton

Matthew Bursnall

Mike Bradburn

Jennifer Petrie

Lizzie Swaby

Gemma Hackney

Judith Cohen
